# Supplementary material for: KLF4 recruits SWI/SNF to increase chromatin accessibility and reprogram the endothelial enhancer landscape under laminar shear stress
Source: Nat Commun. 2022 Aug 23;13:4941. doi: 10.1038/s41467-022-32566-9 (PMC9399231; doi:10.1038/s41467-022-32566-9)
Supplement: Supplementary file 1 — Supplementary Information [file 41467_2022_32566_MOESM1_ESM.pdf]

Supplementary Information

DAR with increased accessibility under LSS

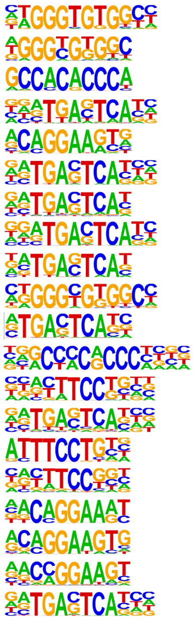

Motif	p-Value	% of DAR
KLF1 (KLF)	1e-279	32.6%
KLF5 (KLF)	1e-227	76.7%
KLF4 (KLF)	1e-227	42.2%
Fra1 (AP1)	1e-208	36.3%
ERG (ETS)	1e-206	76.0%
Atf3 (AP1)	1e-206	40.9%
JunB (AP1)	1e-205	36.7%
Fra2 (AP1)	1e-202	33.7%
BATF (AP1)	1e-196	39.7%
KLF6 (KLF)	1e-196	67.3%
PU.1 (ETS)	1e-193	43.8%
KLF3 (KLF)	1e-189	43.8%
Etv2 (ETS)	1e-177	54.8%
Fosl2 (AP1)	1e-175	26.1%
EWS:ERG (ETS)	1e-167	44.6%
Fli1 (ETS)	1e-164	56.3%
EWS:FLI1 (ETS)	1e-151	36.8%
ETS1 (ETS)	1e-150	55.7%
ETV1 (ETS)	1e-144	65.4%
cJUN (AP1)	1e-141	19.9%

DAR with decreased accessibility under LSS

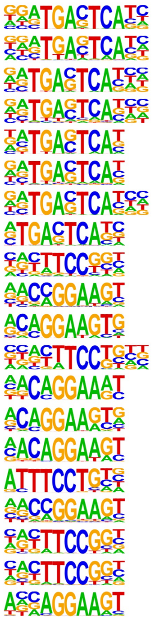

Motif	p-Value	% of DAR
Fra2 (AP1)	1e-211	48.1%
Fra1 (AP1)	1e-209	53.9%
Atf3 (AP1)	1e-205	58.5%
Fosl2 (AP1)	1e-199	36.4%
BATF (AP1)	1e-196	57.5%
JunB (AP1)	1e-195	51.6%
cJun (AP1)	1e-186	29.4%
PU.1 (ETS)	1e-170	58.2%
Fli1 (ETS)	1e-169	63.8%
ETV1(ETS)	1e-154	70.9%
ETS1(ETS)	1e-144	61.9%
Etv2 (ETS)	1e-143	60.4%
EWS:FLI1 (ETS)	1e-141	44.0%
ERG (ETS)	1e-135	77.7%
Ets1-distal (ETS)	1e-130	30.4%
EWS:ERG (ETS)	1e-128	58.9%
GABPA (ETS)	1e-123	52.4%
Elk4 (ETS)	1e-104	32.1%
Elk1 (ETS)	1e-98	31.5%
ELF3 (ETS)	1e-96	1.9 %

Supplementary Figure 1. Motif enrichment of differentially accessible regions (DAR) with increased or decreased accessibility with laminar shear stress (LSS).
Data were analyzed using Homer. Lists show the top 20 most enriched motifs. *P* values were determined by binomial test.

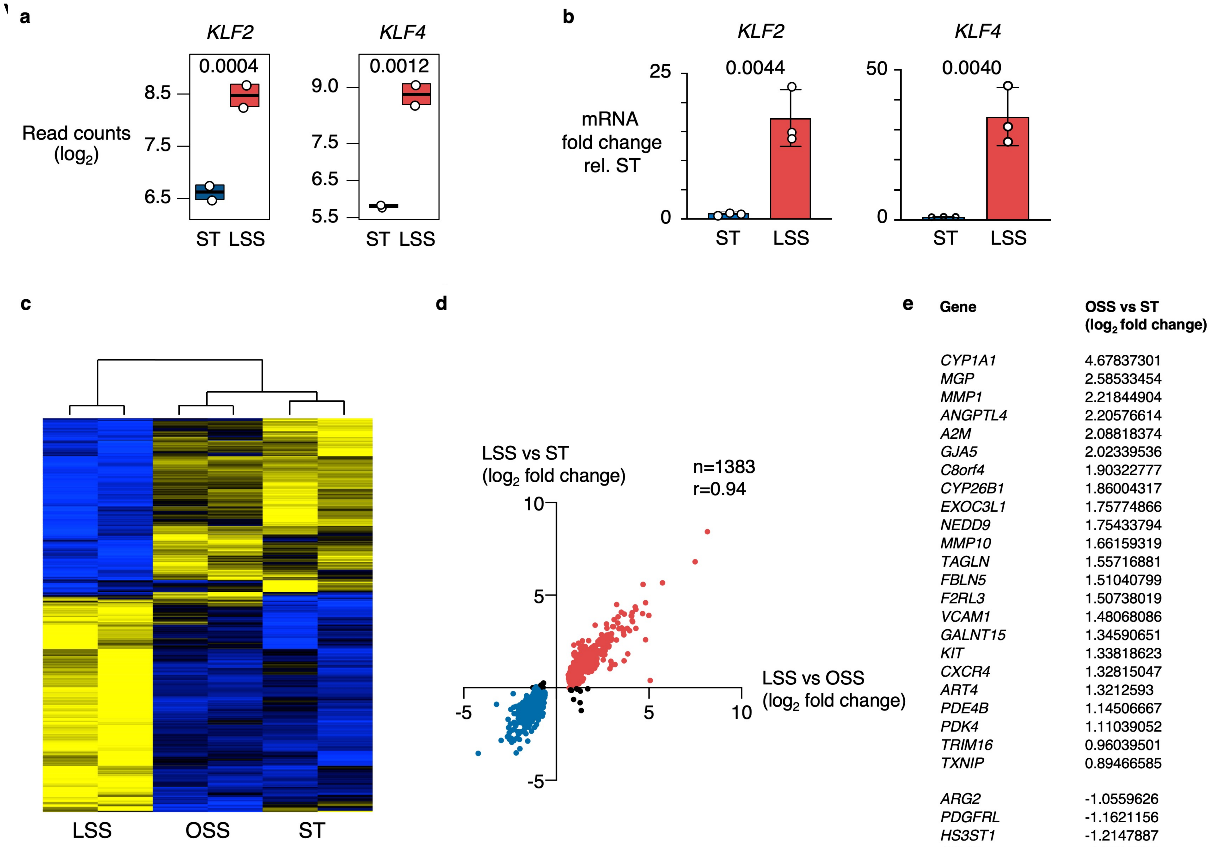

e

Gene	OSS vs ST (log$_2$ fold change)
CYP1A1	4.67837301
MGP	2.58533454
MMP1	2.21844904
ANGPTL4	2.20576614
A2M	2.08818374
GJA5	2.02339536
C8orf4	1.90322777
CYP26B1	1.86004317
EXOC3L1	1.75774866
NEDD9	1.75433794
MMP10	1.66159319
TAGLN	1.55716881
FBLN5	1.51040799
F2RL3	1.50738019
VCAM1	1.48068086
GALNT15	1.34590651
KIT	1.33818623
CXCR4	1.32815047
ART4	1.3212593
PDE4B	1.14506667
PDK4	1.11039052
TRIM16	0.96039501
TXNIP	0.89466585
ARG2	-1.0559626
PDGFRL	-1.1621156
HS3ST1	-1.2147887
ACP5	-1.3702734
FAM167B	-1.4663347
MAGED4B	-1.5601674
RP11-498C9.3	-2.0118731
RP11-449P15.1	-2.3423068

Supplementary Figure 2. *KLF2* and *KLF4* expression is increased under laminar shear stress (LSS) versus static (ST), and PAEC cultured under ST conditions show similar gene expression changes compared to those exposed to oscillatory shear stress (OSS). **a**. Box plot depicting the normalized read counts for KLF2 and KLF4 obtained by RNA-Seq from PAEC exposed to 15 dyn/cm^2 of LSS for 24 h versus ST. Data are representative of two independent experiments. Statistical significance was determined using a 10% FDR threshold. Middle bar represents the median. **b**. Bar graphs showing confirmation by RT-qPCR of the induction of KLF2 and KLF4 upon exposure to 15 dyn/cm^2 of LSS for 24 h, normalized to ST control levels. Data are shown as the mean ± s.e.m. Data are representative of three independent experiments. *P* values were determined by Student's two-tailed *t*-test. Source data are provided as a Source Data file. **c**. Heatmap showing the differentially expressed genes (DEG) under LSS versus OSS and ST. **d**. Scatterplot showing the correlation between changes in gene expression under LSS versus ST, with LSS versus OSS. Values were calculated by a two-tailed Pearson R test. **e**. List of the genes that were differentially expressed under OSS versus ST.

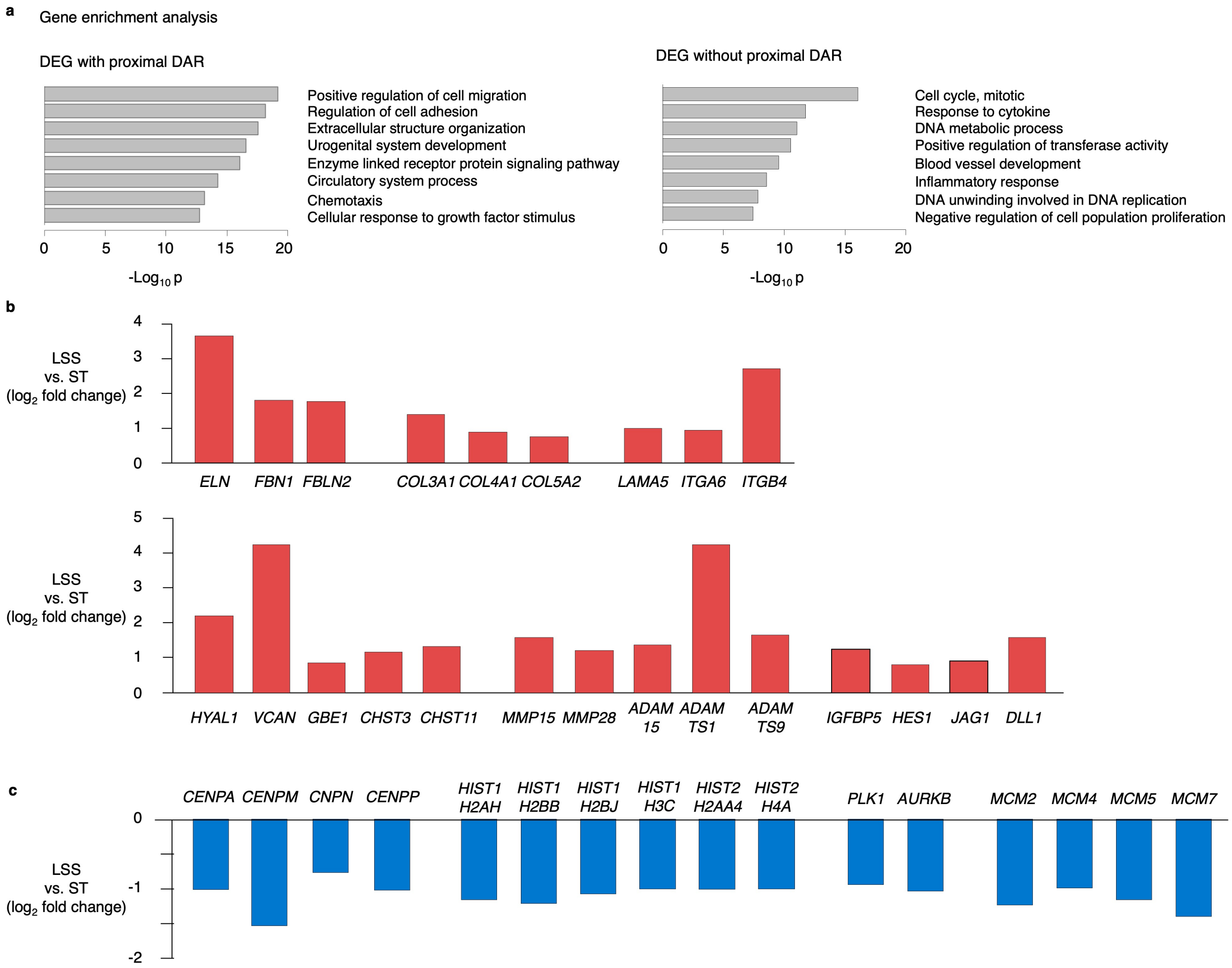

Supplementary Figure 3. Gene expression changes of genes, in enriched pathways, with or without proximal accessibility changes.

a. Gene enrichment analyses using Metascape of differentially expressed genes (DEG) that have proximal accessibility changes (DAR) (left panel) and those without (right panel). *P* values were determined by a one-tailed hypergeometric test with Benjamin-Hochberg adjustment. **b.** Bar graphs showing gene expression changes of DEG that have proximal accessibility changes in PAEC exposed to 15 dyn/cm^2 of LSS for 24 h versus ST. **c**. Bar graphs showing gene expression changes for DEG that do not have proximal accessibility changes under LSS versus ST. Statistical significance was determined using a 10% FDR threshold.

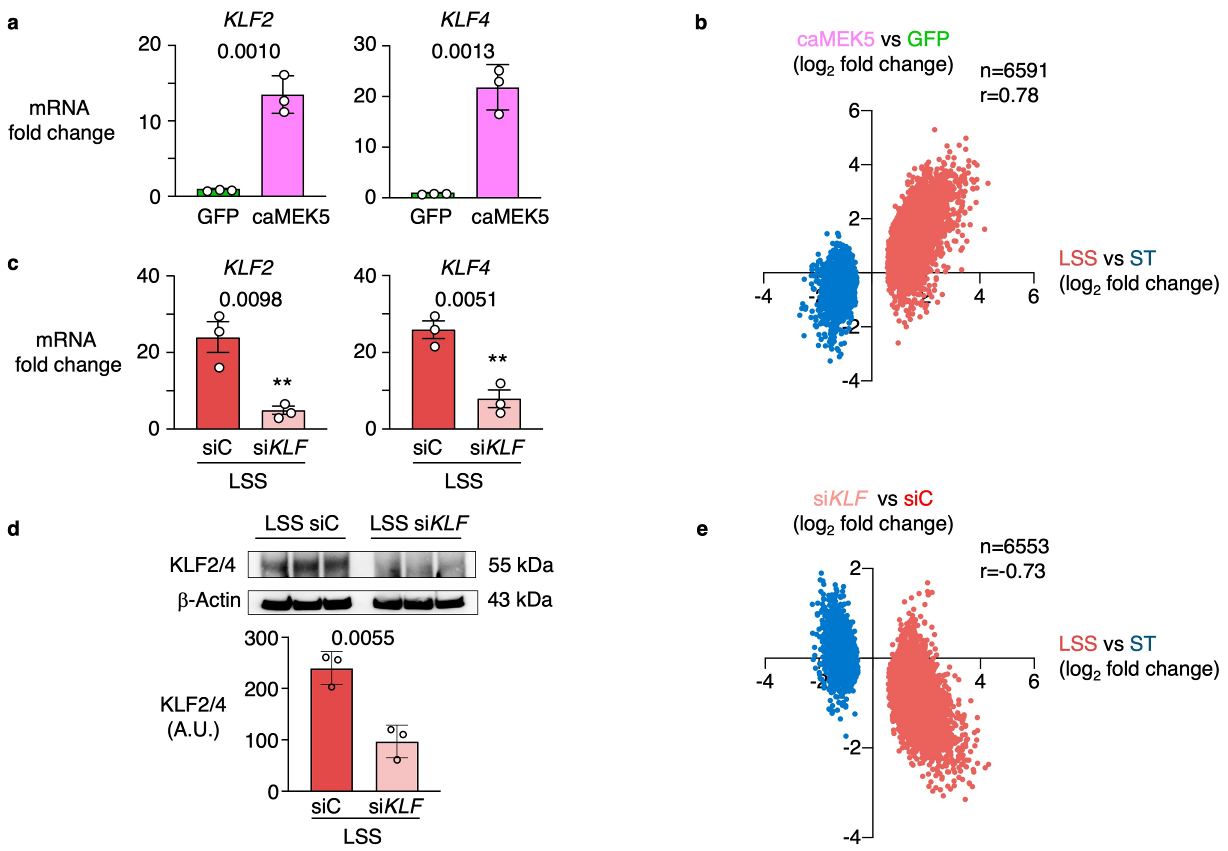

Supplementary Figure 4. KLF2 and KLF4 expression with gain- and loss-of-function, and correlation with accessibility changes observed in wildtype cells exposed to LSS.

a. Bar graphs showing the expression of *KLF2* and *KLF4* by RT-qPCR in PAEC transduced with adenoviral constructs encoding caMEK5, normalized to GFP control. Data are representative of three independent experiments. *P* values were determined by Student's two-tailed *t*-test. **b**. Scatterplot showing the correlation of all LSS vs ST DAR with accessibility changes in caMEK5-transduced PAEC at those sites. **c**. Bar graphs showing the expression of *KLF2* and *KLF4* by RT-qPCR in PAEC with RNAi targeting *KLF2* and *KLF4* (si*KLF*) versus non-targeting negative controls (siC), normalized to Static siC. Data are representative of three independent experiments. *P* values were determined by Student's two-tailed *t*-test. **d**. Western blot analysis of KLF2/4 protein expression in PAEC with RNAi targeting *KLF2* and *KLF4* (si*KLF*) versus non-targeting negative controls (siC), normalized to LSS siC. Data are representative of three independent experiments. *P* values were determined by Student's two-tailed *t*-test. **e**. Scatterplot showing the correlation of all LSS vs ST DAR with accessibility changes in PAEC treated with RNAi targeting *KLF2* and *KLF4* at those sites. For **a**, **c** and **d**, source data are provided as a Source Data file.

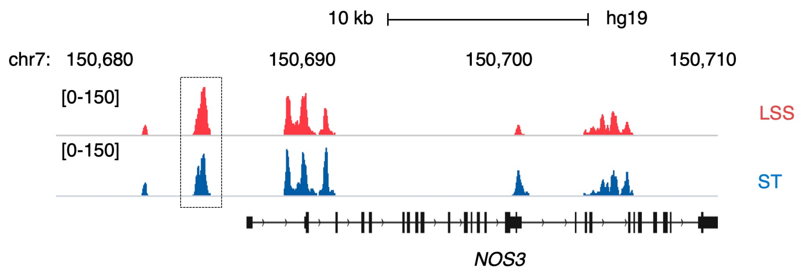

Supplementary Figure 5. ATAC-Seq tracks showing accessibility at the *NOS3* gene promotor under LSS.

ATAC-Seq tracks show increased accessibility at the promotor region of *NOS3* in PAEC exposed to 15 dyn/cm^2 of LSS for 24 h versus ST.

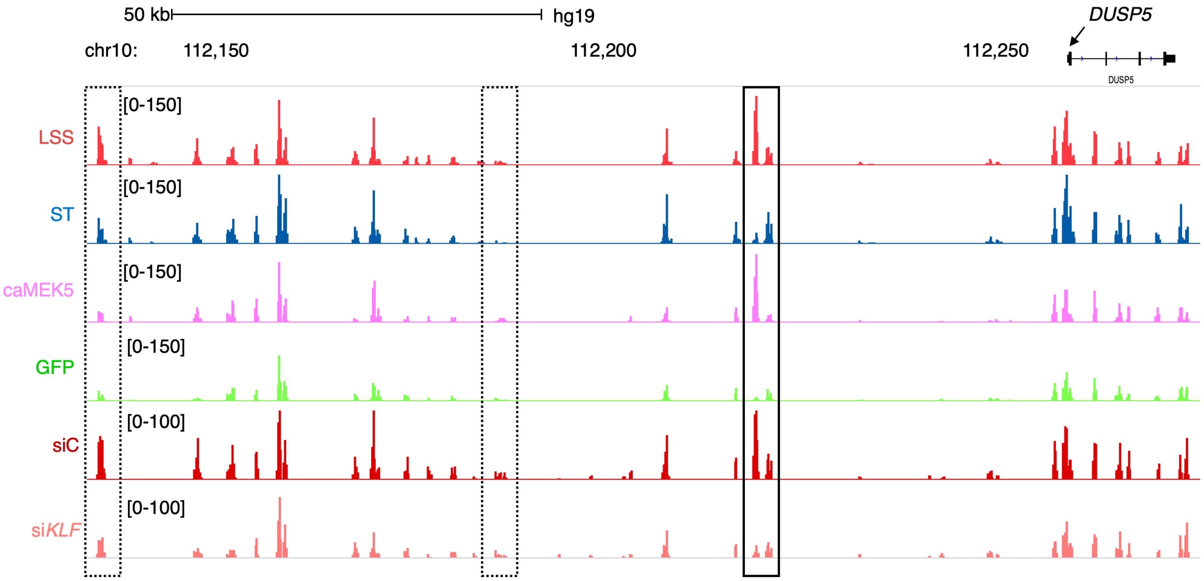

Supplementary Figure 6. ATAC-Seq tracks showing differentially accessible regions at the *DUSP5* locus.

ATAC-Seq tracks showing sites with increased accessibility proximal to the *DUSP5* TSS in PAEC exposed to 15 dyn/cm^2 of LSS for 24 h versus ST. Highlighted regions are differentially accessible under LSS versus ST, with the region framed by the solid line indicating the DAR described in the main text.

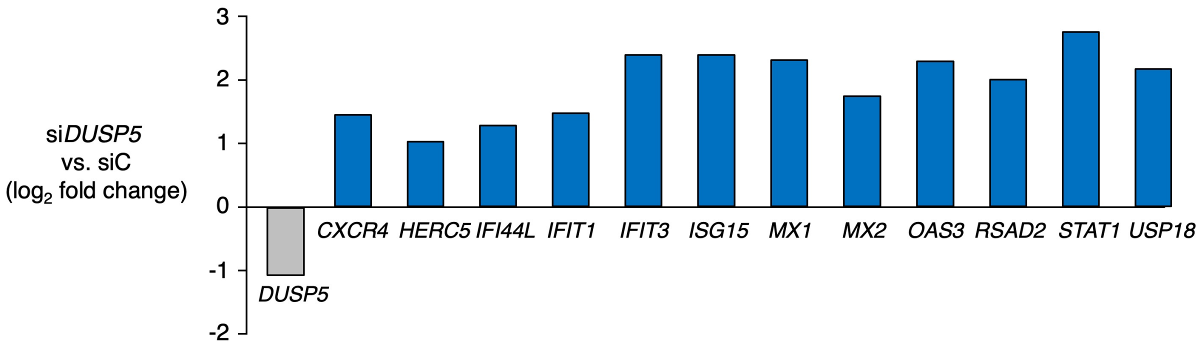

Supplementary Figure 7. Validation of *DUSP5* target genes.
Bar graphs from RNA-Seq data indicating de-repression of genes related to an antiviral response and activation of the interferon signaling pathway in PAEC treated with *DUSP5* siRNA prior to exposure to LSS. Data are representative of two independent experiments. Statistical significance was determined using 10% FDR threshold.

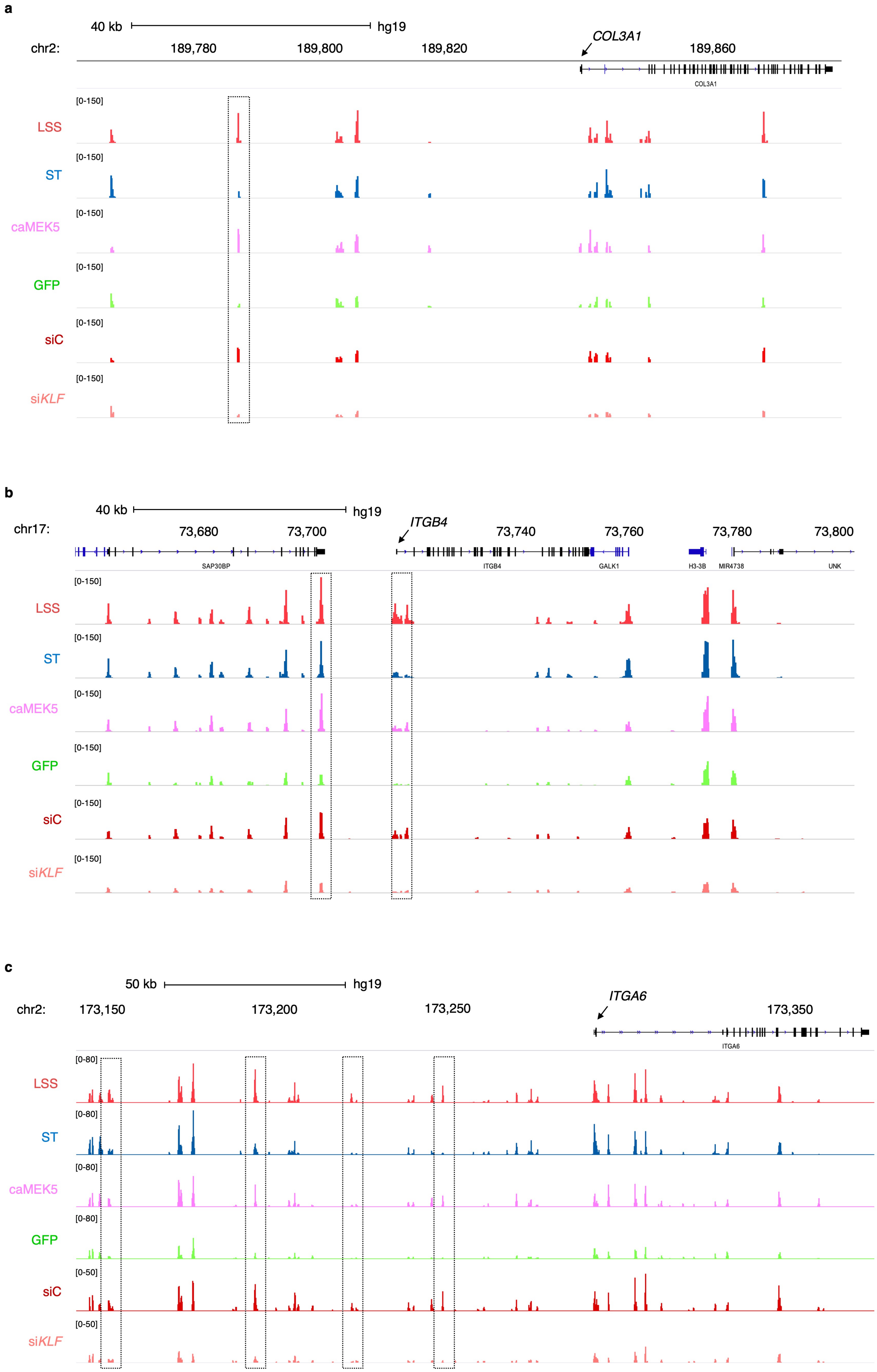

a
40 kb
hg19
chr2:
189,780
189,800
189,820
189,860
COL3A1
[0-150]
LSS
ST
caMEK5
GFP
siC
siKLF
b
40 kb
hg19
chr17:
73,680
73,700
73,740
73,760
73,780
73,800
ITGB4
SAP30BP
GALK1
H3-3B
MIR4738
UNK
[0-150]
LSS
ST
caMEK5
GFP
siC
siKLF
c
50 kb
hg19
chr2:
173,150
173,200
173,250
173,350
ITGA6
[0-80]
[0-50]
LSS
ST
caMEK5
GFP
siC
siKLF

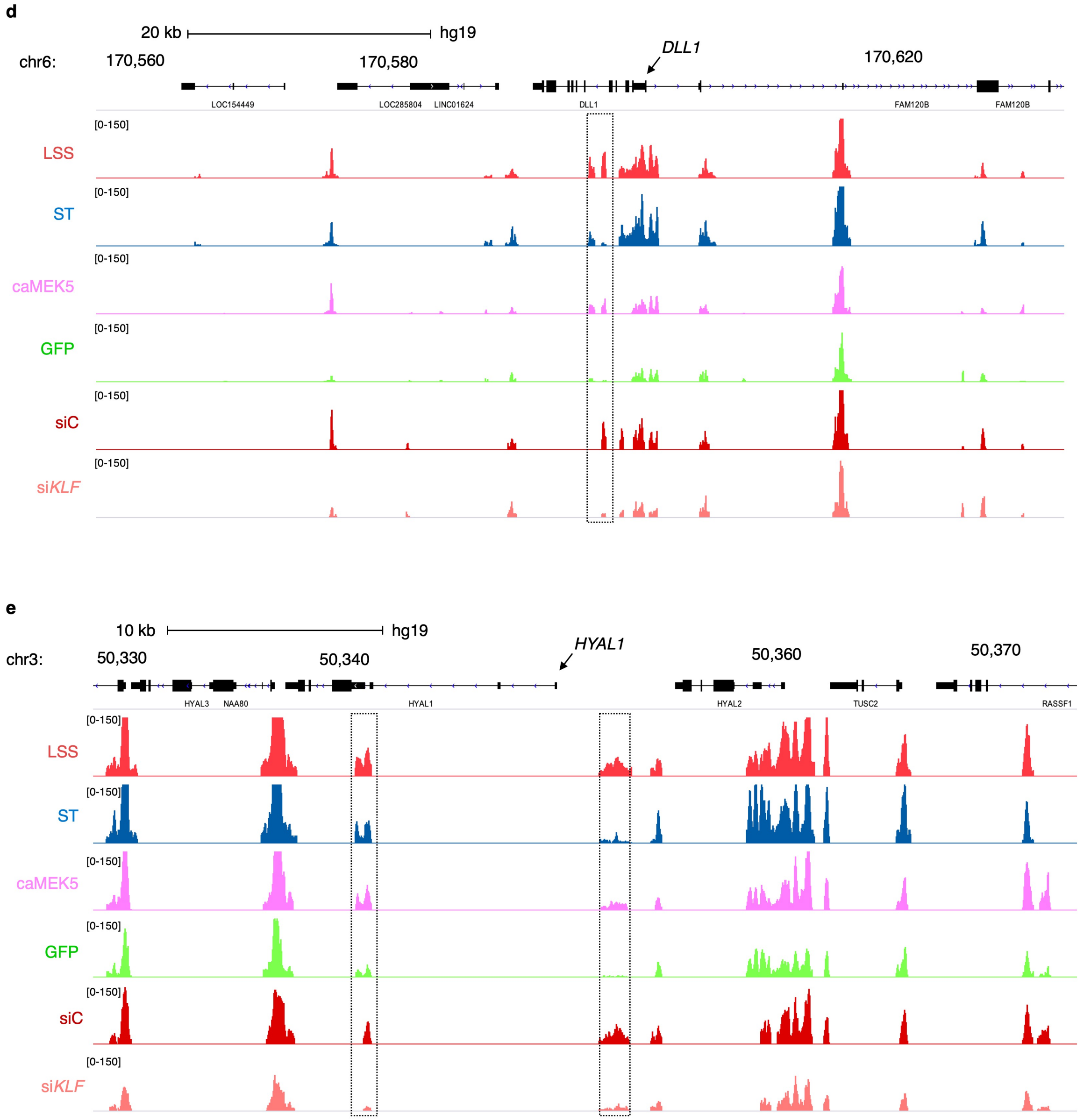

Supplementary Figure 8. ATAC-Seq tracks showing chromatin accessibility changes under LSS versus ST, and with KLF gain- or loss-of-function.

ATAC-Seq tracks showing accessibility at **a.** The *COL3A1* locus. **b.** The *ITGB4* locus. **c.** The *ITGA6* locus. **d.** The *DLL1* locus. **e.** The *HYAL1* locus. For all examples, top rows show untreated PAEC exposed to 15 dyn/cm^2 of LSS for 24 h (red) or static culture conditions (blue); middle rows show KLF gain-of-function in PAEC transduced with adenoviral vectors encoding caMEK5 (pink) or GFP as control (green); bottom rows show KLF loss-of-function using siRNA targeting *KLF2* and *KLF4* (si*KLF*; light red), or non-targeting controls (siC; red).

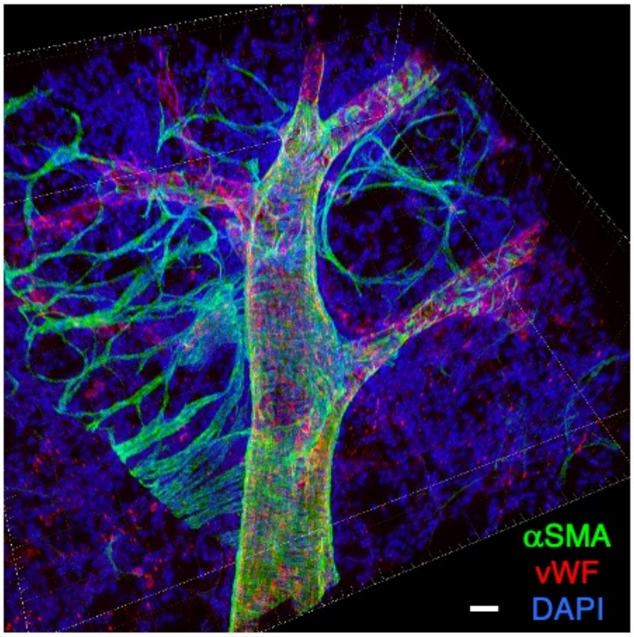

Supplementary Figure 9. Representative image of a 3D reconstruction of a rat pulmonary artery.

Smooth muscle alpha-2 actin (αSMA, green); von Willebrand factor (vWF, red); DAPI, blue. Scale bar, 30 μm**.**

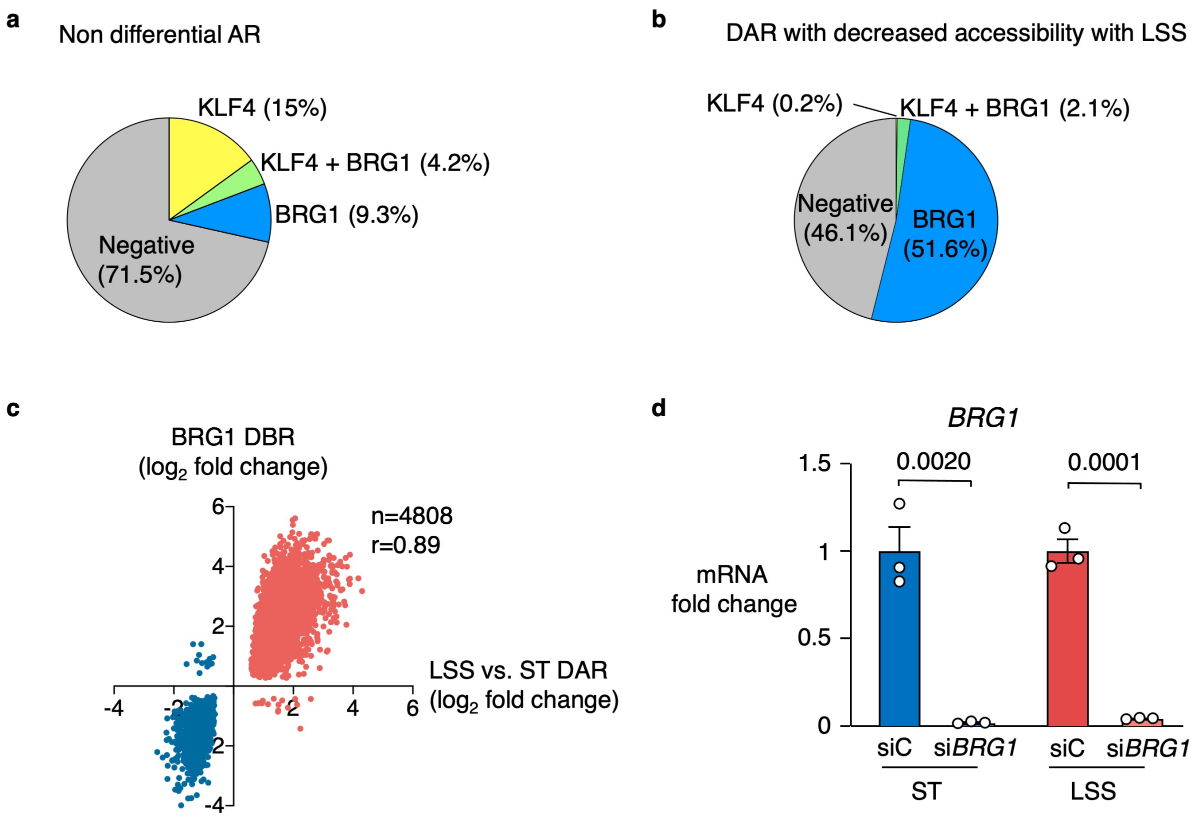

Supplementary Figure 10. KLF4 and BRG1 co-occupancy at accessible regions (AR), and at DAR with decreased accessibility under LSS; correlation between BRG1 occupancy and accessibility changes; and validation of BRG1 loss-of-function.

a. Pie chart showing the percentage of regions that are accessible under both LSS and ST, that are differentially enriched for KLF4 and/or BRG1. **b.** Pie chart showing enrichment for KLF4 and BRG1 in regions that have a decreased accessibility with LSS. **c.** Scatterplot showing the correlation between BRG1 differential binding regions (DBR) and accessibility changes at LSS vs ST DARs. r=0.89 with p<0.0001 calculated using a two-tailed Pearson R test. **d.** Bar graphs showing the expression of BRG1 in PAEC treated with siRNA targeting *BRG1* (si*BRG1*) versus non-targeting negative controls (siC) under LSS versus ST, normalized to Static siC. Data are shown as the mean +/- s.e.m. Data are representative of three independent experiments. *P* values were determined by Student's two-tailed *t*-test. Source data are provided as a Source Data file.

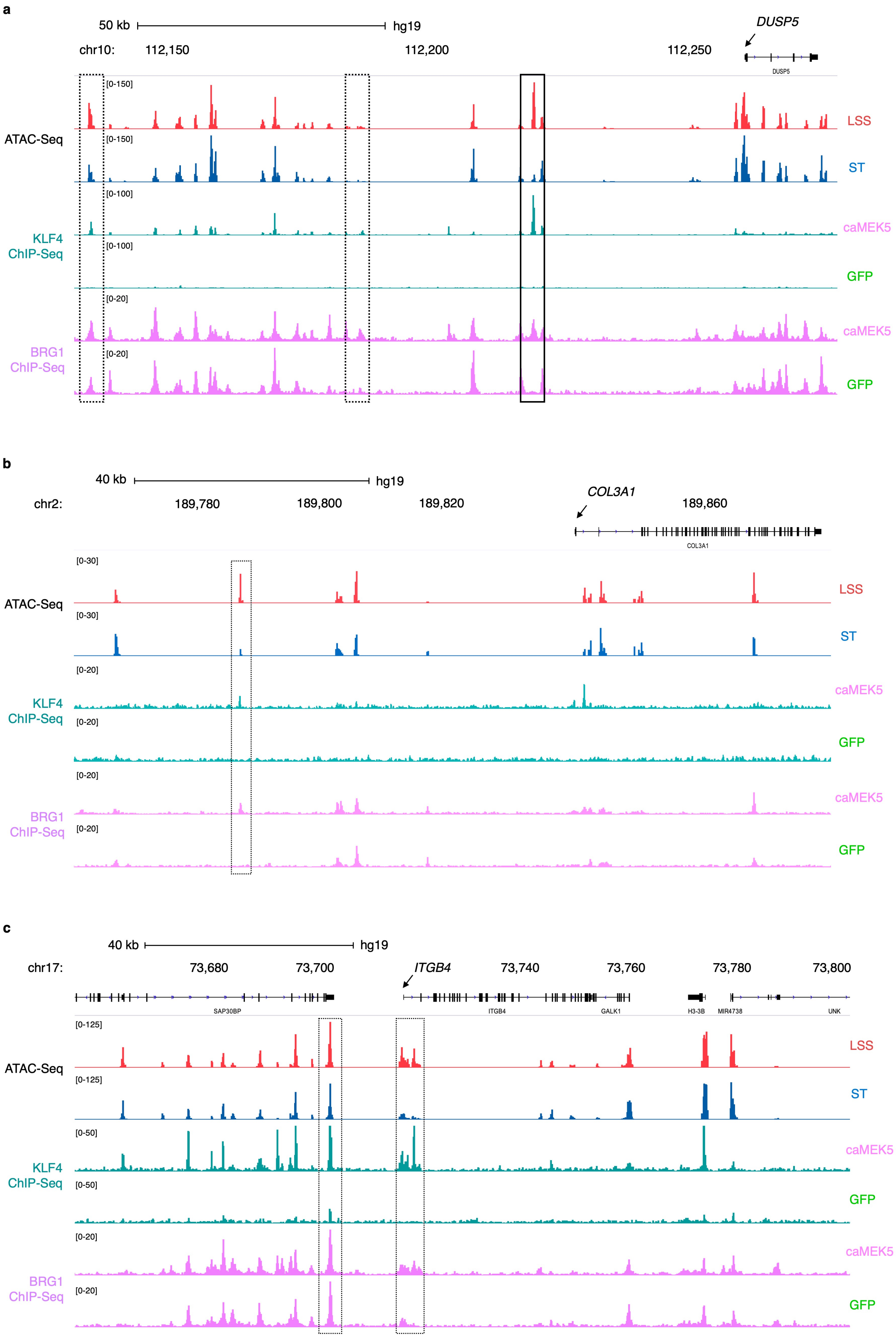

a
50 kb
hg19
chr10:
112,150
112,200
112,250
DUSP5
[0-150]
[0-100]
[0-20]
ATAC-Seq
KLF4
ChIP-Seq
BRG1
ChIP-Seq
LSS
ST
caMEK5
GFP
b
40 kb
hg19
chr2:
189,780
189,800
189,820
189,860
COL3A1
[0-30]
[0-20]
ATAC-Seq
KLF4
ChIP-Seq
BRG1
ChIP-Seq
LSS
ST
caMEK5
GFP
c
40 kb
hg19
chr17:
73,680
73,700
73,740
73,760
73,780
73,800
ITGB4
SAP30BP
GALK1
H3-3B
MIR4738
UNK
[0-125]
[0-50]
[0-20]
ATAC-Seq
KLF4
ChIP-Seq
BRG1
ChIP-Seq
LSS
ST
caMEK5
GFP

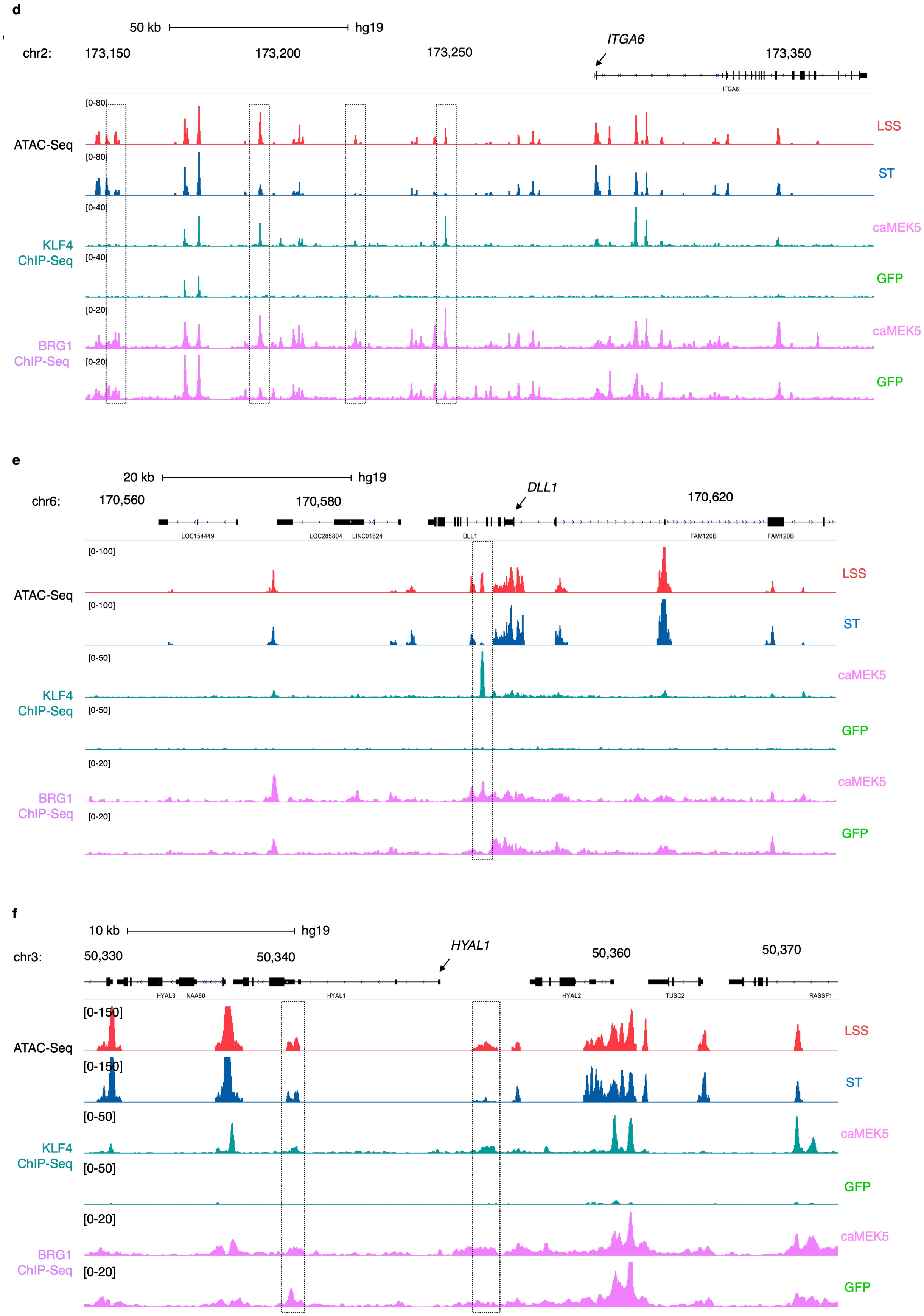
d
50 kb
hg19
chr2:
173,150
173,200
173,250
ITGA6
173,350
ITGA6
[0-80]
ATAC-Seq
LSS
[0-80]
ST
[0-40]
KLF4
ChIP-Seq
caMEK5
[0-40]
GFP
[0-20]
BRG1
ChIP-Seq
caMEK5
[0-20]
GFP
e
20 kb
hg19
chr6:
170,560
170,580
DLL1
170,620
LOC154449
LOC285804
LINC01624
DLL1
FAM120B
FAM120B
[0-100]
ATAC-Seq
LSS
[0-100]
ST
[0-50]
KLF4
ChIP-Seq
caMEK5
[0-50]
GFP
[0-20]
BRG1
ChIP-Seq
caMEK5
[0-20]
GFP
f
10 kb
hg19
chr3:
50,330
50,340
HYAL1
50,360
50,370
HYAL3
NAA80
HYAL1
HYAL2
TUSC2
RASSF1
[0-150]
ATAC-Seq
LSS
[0-150]
ST
[0-50]
KLF4
ChIP-Seq
caMEK5
[0-50]
GFP
[0-20]
BRG1
ChIP-Seq
caMEK5
[0-20]
GFP

Supplementary Figure 11. ATAC-Seq and KLF4 and BRG1 ChIP-Seq tracks showing enrichment for both factors at DAR of candidate genes.

a. The *DUSP5* locus **b.** The *COL3A1* locus. **c.** The *ITGB4* locus. **d.** The *ITGA6* locus. **e.** The *DLL1* locus. **f.** The *HYAL1* locus. For all examples, top rows show untreated PAEC exposed to 15 dyn/cm^2 of LSS for 24 h (red) or static culture conditions (blue); for KLF4 (green) and BRG1 (pink) ChIP-Seq, PAEC were transduced with adenoviral vectors encoding caMEK5 or GFP as control. Highlighted regions are differentially accessible under LSS versus ST. In **a,** the region framed by the solid line indicates the DAR 39 kb upstream of *DUSP5* described in the main text.

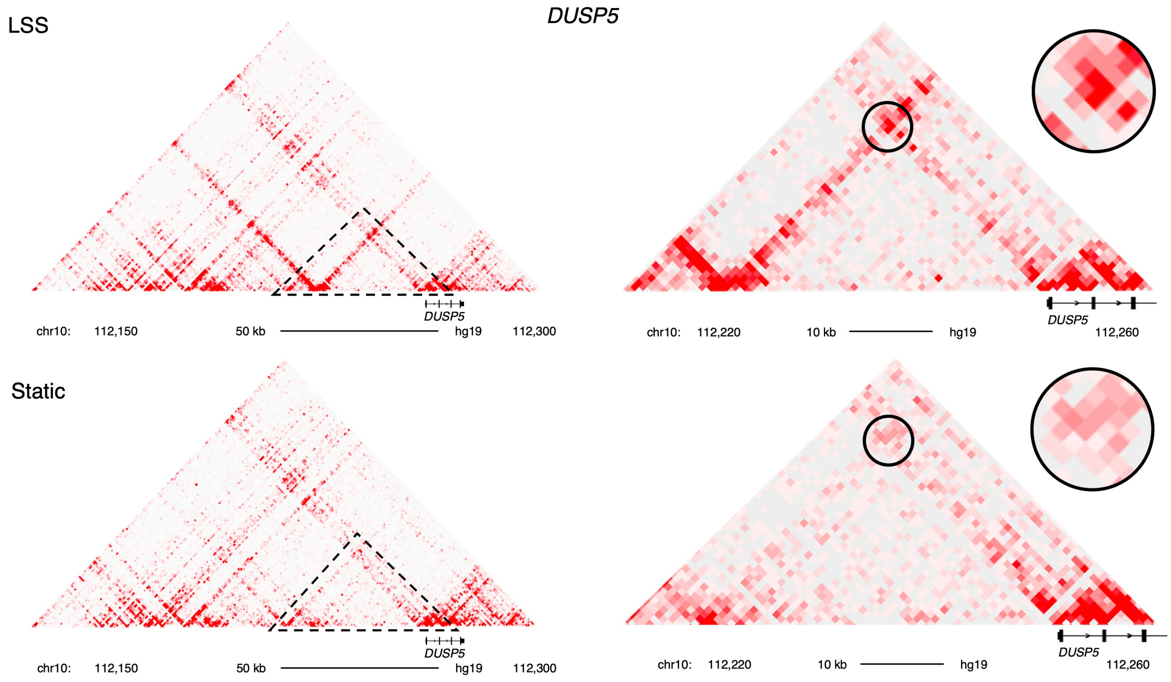

Supplementary Figure 12. HiCHIP contact map at the *DUSP5* locus.
H3K27ac HiChIP contact map showing enrichment for contacts at the *DUSP5* locus in PAEC exposed to 15 dyn/cm^2 of LSS for 24 h versus ST. Circles highlight the enhancer-promotor contact described in the main text.

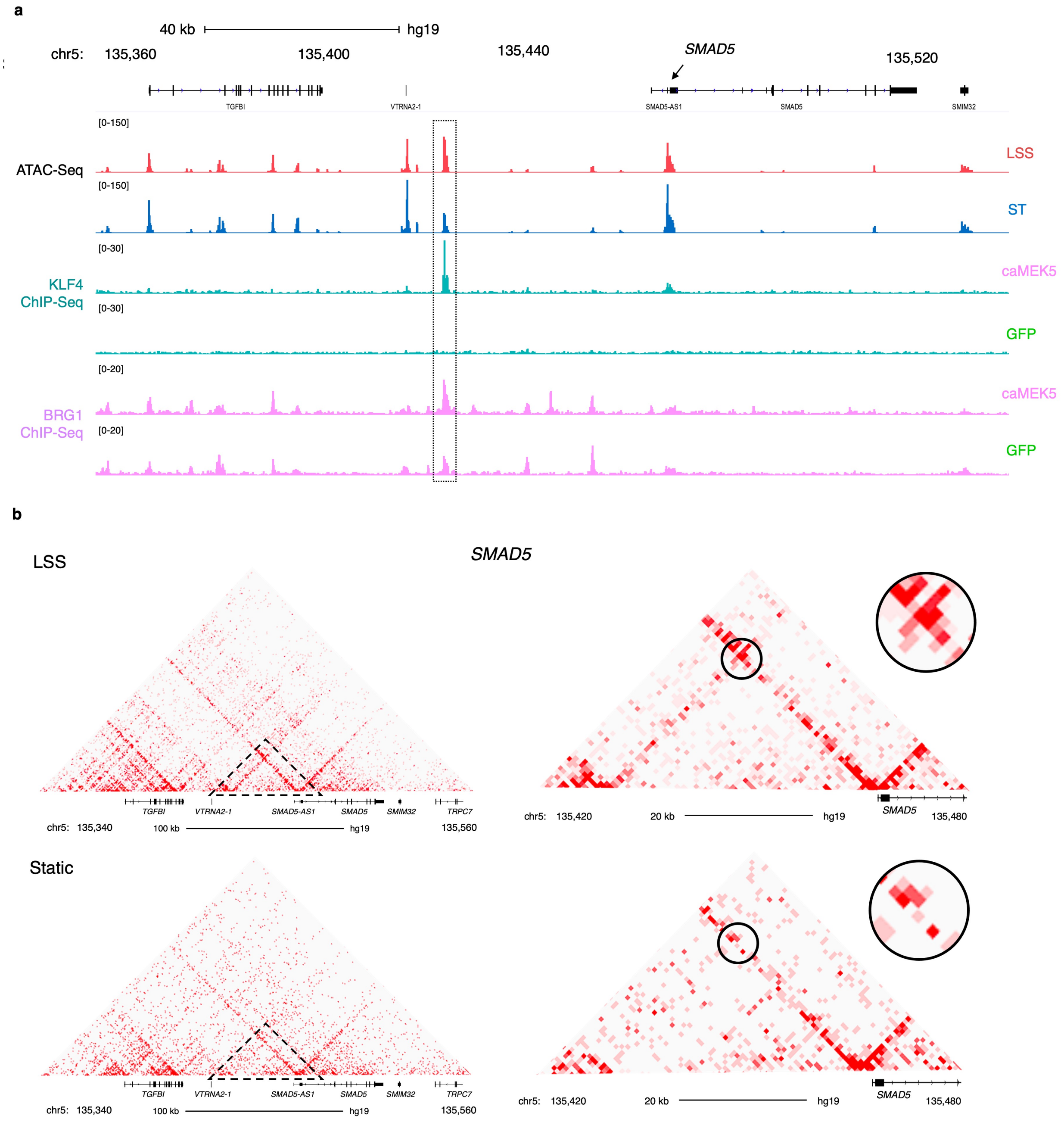

Supplementary Figure 13. ATAC-Seq, KLF4 and BRG1 ChIP-Seq tracks, and H3K27ac HiChIP contact map at the *SMAD5* locus.

a. Top rows show ATAC-Seq tracks of untreated PAEC exposed to 15 dyn/cm^2 of LSS for 24 h (red) or static culture conditions (blue). Middle and bottom, ChIP-Seq tracks for KLF4 (teal) and BRG1 (pink) in PAEC that were transduced with adenoviral vectors encoding caMEK5 or GFP as control. Highlighted region is differentially accessible under LSS versus ST. **b.** H3K27ac HiChIP contact map showing enrichment for contacts at the *SMAD5* locus in PAEC exposed to 15 dyn/cm^2 of LSS for 24 h versus ST. Circles highlight the enhancer-promotor contact described in the main text

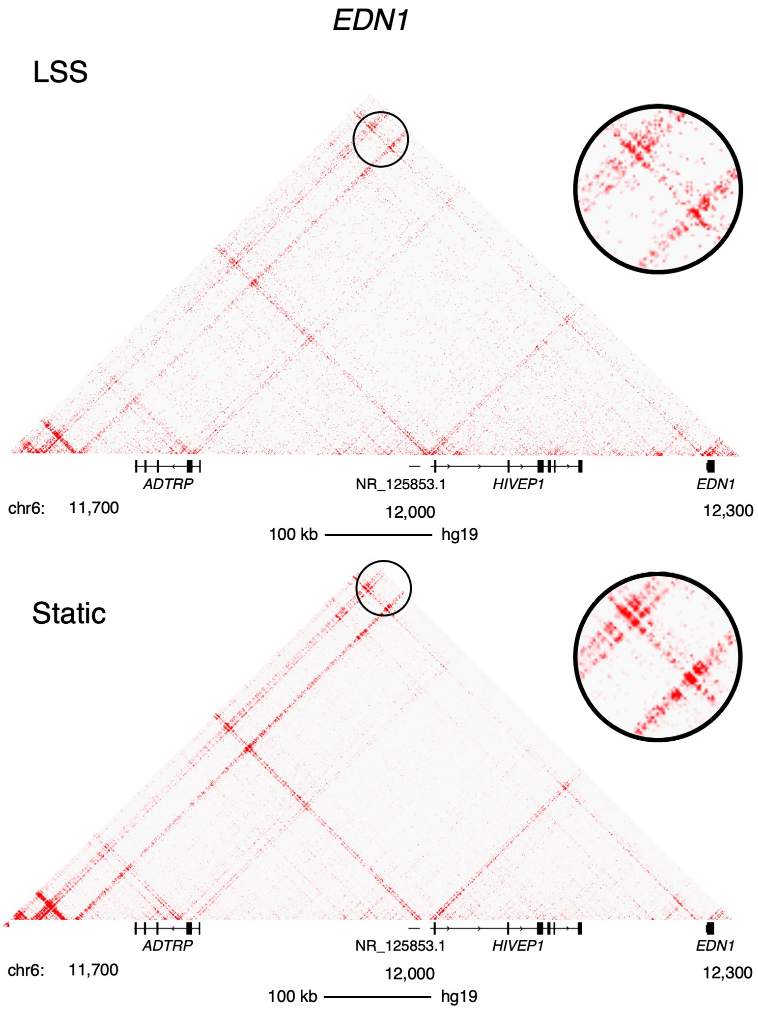

Supplementary Figure 14. HiCHIP contact map at the *EDN1* locus.

H3K27ac HiChIP contact map showing enrichment for contacts at the *EDN1* locus in PAEC exposed to 15 dyn/cm^2 of LSS for 24 h versus ST. Circles highlight the enhancer-promotor contact described in the main text.

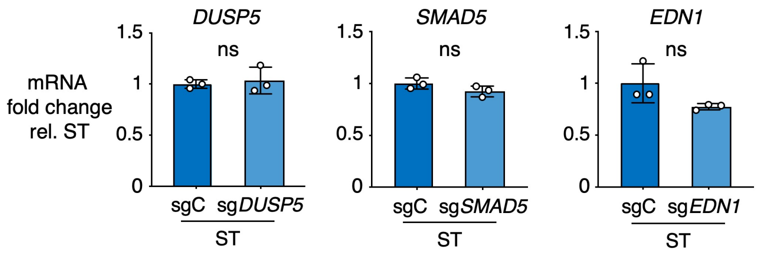

Supplementary Figure 15. CRISPRi of candidate enhancers under ST conditions.
Bar graphs showing the expression of *DUSP5*, *SMAD5* and *EDN1* in PAEC treated with gRNA targeting the candidate enhancer regions under ST conditions. Data are shown as the mean +/- s.e.m. Data show three independent experiments. Changes were not significant (ns), calculated using Student's two-tailed *t*-test. Source data are provided as a Source Data file.

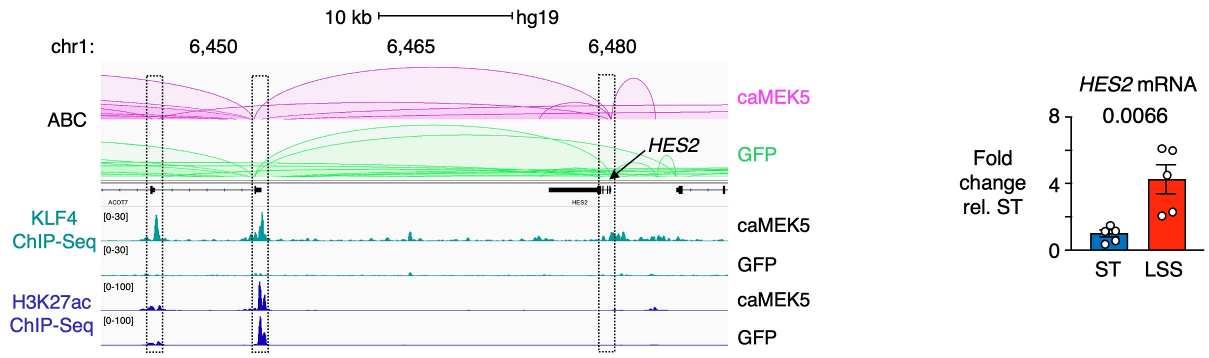

Supplementary Figure 16. Proximal enhancers targeting *HES2* identified by ABC.
ABC and ChIP-Seq tracks illustrating a KLF4 bound enhancer loop that is predicted to target *HES2*. *HES2* mRNA was determined by RT-qPCR in PAEC exposed to 15 dyn/cm^2 of LSS vs ST conditions for 24 h, and shown normalized to ST expression levels (right panel). Data are shown as the mean +/- s.e.m. Data are representative of three independent experiments. *P* value was determined by Student's two-tailed *t*-test. Source data are provided as a Source Data file.

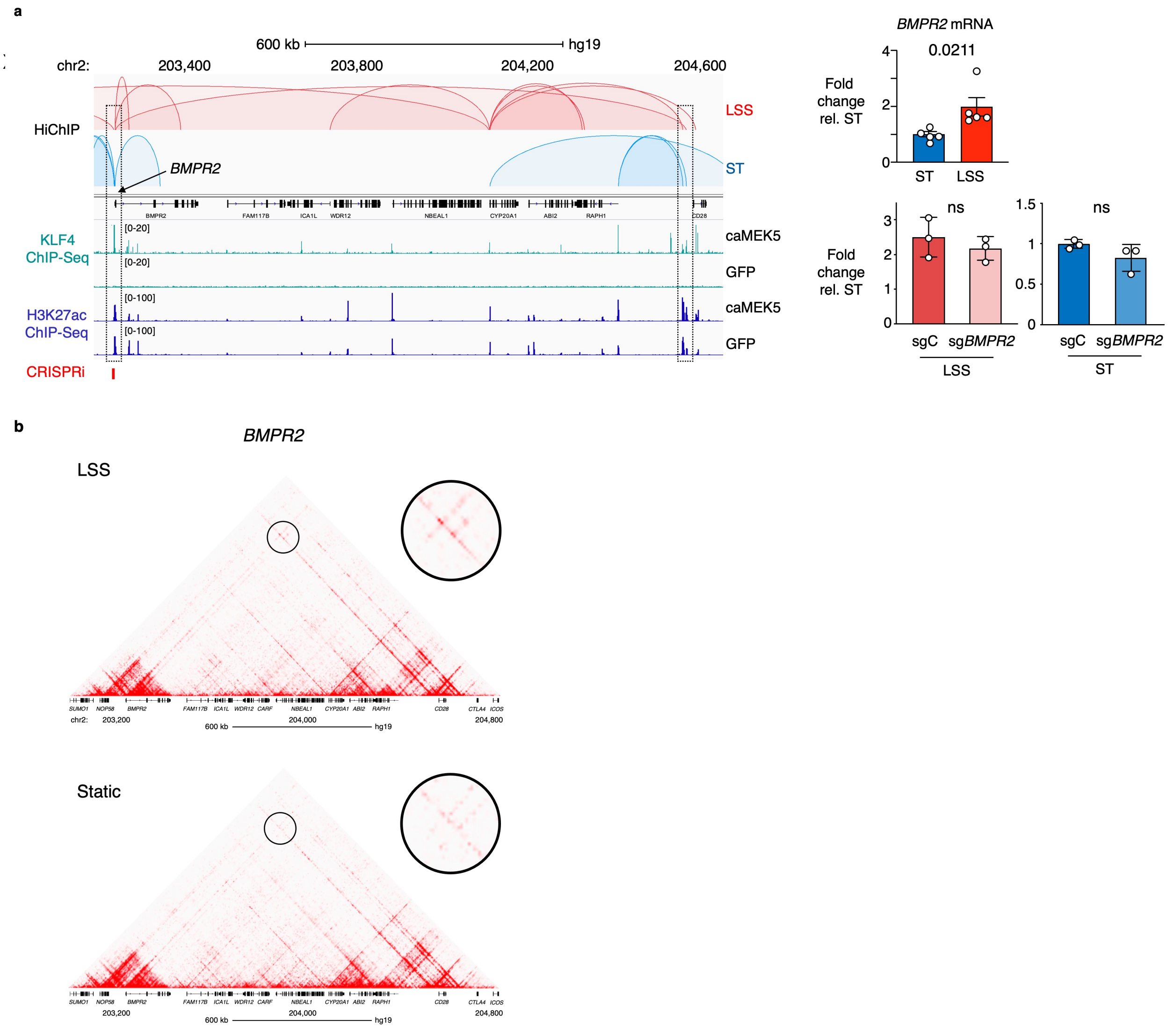

Supplementary Figure 17. Distal enhancer targeting *BMPR2* identified by HiChIP.
a. HiChIP and KLF4 ChIP-Seq tracks illustrating a KLF4 bound enhancer loop that targets *BMPR2*. *BMPR2* mRNA was determined by RT-qPCR in PAEC exposed to 15 dyn/cm^2 of LSS vs ST conditions for 24 h, and shown normalized to ST expression levels (upper right panel). CRISPRi targeting the enhancer site did not result in a significant decrease in *BMPR2* gene expression under LSS or ST (lower right panel). Data are shown as the mean +/- s.e.m. Data are representative of three independent experiments. *P* values were determined by Student's two-tailed *t*-test. ns: not significant. Source data are provided as a Source Data file. **b**. H3K27ac HiChIP contact maps showing enrichment for contacts in regions surrounding *BMPR2*. Circles highlight the enhancer-promotor contact described.

a

Variant	Locus (hg19)	Target gene(s)
rs2065152	chr1:90228519	*LRRC8B; LRRC8C-DT*
rs3774702	chr3:63856870	*THOC7*
rs62270945	chr3:128201889	*GATA2*
rs1706003	chr3:194299967	*TMEM44-AS1*
Rs13112725	chr4:106911742	*INTS12; DKK2*
rs114503346	chr5:172192350	*SH3PXD2B; ERGIC1; LOC100268168; RPL26L1*
rs17080102	chr6:151004770	*PLEKHG1*
rs10233127	chr7:30933453	*MINDY4; AQP1*
rs1011018	chr7:13943264	*HIPK2; TBXAS1*
rs891511	chr7:150704843	*NOS3*
rs3918226	chr7:150690176	*AGAP3*
rs1870735	chr7:155744303	*SHH; LOC3896*
rs28558845	chr9:4334791	*GLIS3; SLC1A*
rs7126805	chr11: 828916	*SLC25A22; RPLP2*
rs67330701	chr11:69079707	*MYEOV*
rs11222386	chr11:130779068	*LINC02551*
rs73099903	chr12:53440779	*EIF4B; SOAT2*
rs17608766	chr17:45013271	*GOSR2; WNT9B*
rs12474050	chr2:220362557	*GMPPA*
rs3737801	chr1:27960832	*FGR; AHDC1*
rs9337951	chr10:30317073	*JCAD*
rs6021247	chr20:50108980	*NFATC2*

b

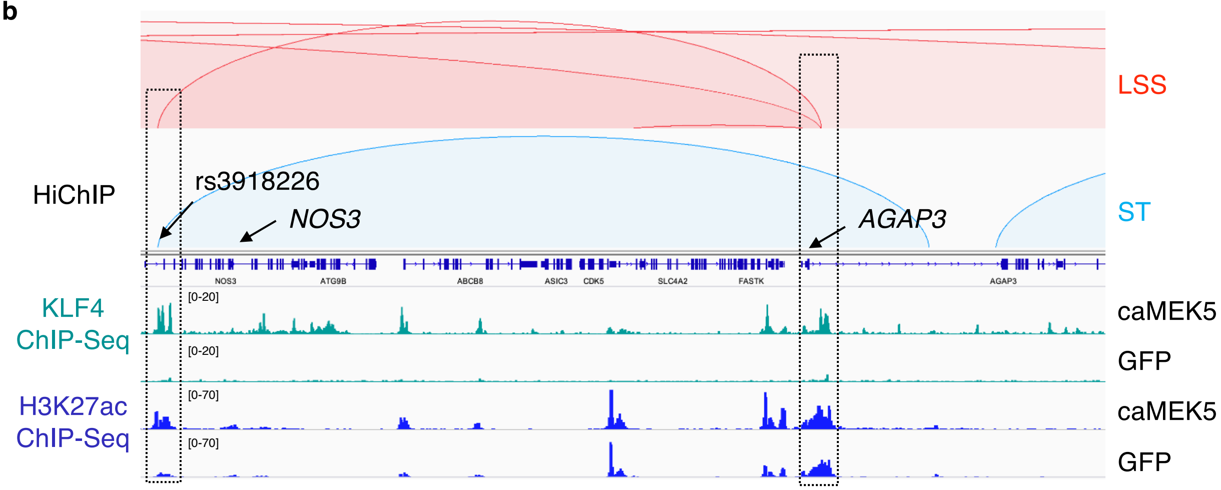

Supplementary Figure 18. Variants associated with blood pressure traits are in KLF4-bound enhancer elements.

a. Variants associated with blood pressure traits that intersect with KLF4 bound enhancer elements identified by ABC. **B**. HiChIP and ChIP-Seq tracks illustrating that rs3918226 is located in a KLF4 bound enhancer loop that targets *AGAP3*.

Supplementary Table 1. qPCR primers used for RT-qPCR and ATAC-qPCR, and sgRNA sequences used for CRISPRi.

RT-qPCR primers

Gene	Forward	Reverse
BMPR2	CTGCGGCTGCTTCGCAGAAT	TGGTGTTGTGTCAGGAGGTGG
DUSP5	ACAAATGGATCCCTGTGGAA	CCTCCCTTTTCCCTGACAC
EDN1	ACGGAACAACGTGCTCGGGA	AGTGGGTTTCTCCCCGCCGT
HES2	CGCATCAACCAGAGCCTGA	GAGCAGTTGGAGTTCTCCCG
KLF2	CATCTGAAGGCGCATCTG	CGTGTGCTTTCGGTAGTGG
KLF4	GGGAGAAGACACTGCGTCA	GGAAGCACTGGGGGAAGT
SMAD5	TTGCTCAGCTTCTGGCTCAA	CCGGTGATATTCTGCTCCCC
ACTB	CCAACCGCGAGAAGATGA	CCAGAGGCGTACAGGGATAG

ATAC-qPCR primers

Gene	Forward	Reverse
DUSP5	CCTCTGCTTTAAATGCCCGG	TTCTTGCCCCTGTAACCACC
GAPDH	CATCTCAGTCGTTCCCAAAGT	TTCCCAGGACTGGACTGT

sgRNA sequences

Targeted Enhancer	sgRNA sequences
DUSP5	AGGUAUUCAGGAAAAGGGUG
SMAD5	CCAACUGGGAGCUAUCUGCA<br>CUUCUCCCCACCCAAACCAG
EDN1	CAUAGACACACGCACGCCCU<br>AGGGGUCAAACCCAUUUGGA<br>UUUUAGCGAAGUGUGGCCGG
HES2	UGGGCGCGGCAGCGAAUCGG<br>CCCCCGCCCCCAACCCACCC
BMPR2	GGGACUCUAGUUUCCAGUCA
Negative Control	GCACUACCAGAGCUAACUCA

Supplementary Table 2. Final read counts for ATAC-Seq, RNA-Seq, ChIP-Seq and HiCHIP studies.

ATAC-Seq

Sample	Uniquely mapped reads (after removal of mtDNA reads)
LSS Rep A	54,522,236
LSS Rep B	64,741,858
LSS Rep C	65,440,066
ST Rep A	34,700,786
ST Rep B	57,938,926
ST Rep C	111,141,742
caMEK5 Rep A	28,782,964
caMEK5 Rep B	47,264,446
caMEK5 Rep C	38,282,136
GFP Rep A	32,012,358
GFP Rep B	53,257,580
GFP Rep C	41,627,494
LSS siC Rep A	42,446,730
LSS siC Rep B	53,248,578
LSS siC Rep C	46,396,964
LSS siKLF Rep A	58,900,248
LSS siKLF Rep B	39,792,606
LSS siKLF Rep C	38,046,002

RNA-Seq (Total RNA)

Sample	Uniquely mapped reads
LSS Rep A	8,001,259
LSS Rep B	7,132,714
ST Rep A	6,781,161
ST Rep B	5,875,492
caMEK5 Rep A	7,106,184
caMEK5 Rep B	10,945,404
caMEK5 Rep C	6,571,156
GFP Rep A	10,121,249
GFP Rep B	10,939,113
GFP Rep C	7,781,010
LSS siC Rep A	10,444,111
LSS siC Rep B	12,016,714
LSS siC Rep C	8,498,003
LSS siKLF Rep A	9,881,653
LSS siKLF Rep B	13,172,724
LSS siKLF Rep C	10,470,478

RNA-Seq (mRNA)

Sample	Uniquely mapped reads
LSS siC Rep A	10,338,301
LSS siC Rep B	7,041,783
LSS siDUSP5 Rep A	4,863,302
LSS siDUSP5 Rep B	5,524,185

ChIP-Seq

Sample	Uniquely mapped reads
GFP H3K27ac Rep A	18,382,335
GFP H3K27ac Rep B	16,026,322
GFP H3K4me1 Rep A	60,611,880
GFP H3K4me1 Rep B	60,236,844
GFP Input Rep AB	19,214,818
GFP KLF4 Rep A	20,684,930
GFP KLF4 Rep B	18,444,652
caMEK5 H3K27ac Rep A	19,492,426
caMEK5 H3K27ac Rep B	14,702,399
caMEK5 H3K4me1 Rep A	70,643,354
caMEK5 H3K4me1 Rep B	59,337,789
caMEK5 Input Rep AB	24,873,542
caMEK5 KLF4 Rep A	19,922,983
caMEK5 KLF4 Rep B	14,609,090
caMEK5 BRG1 Rep A	57,920,609
caMEK5 BRG1 Rep B	60,473,760
caMEK5 Input Rep A	33,435,378
caMEK5 Input Rep B	39,250,161
GFP BRG1 Rep A	61,217,125
GFP BRG1 Rep B	56,048,362
GFP Input Rep A	32,337,689
GFP Input Rep B	37,234,296

H3K27ac HiChIP

Sample	Uniquely mapped reads
LSS Rep A	206,484,763
LSS Rep B	212,414,726
LSS Rep C	206,051,420
ST Rep A	152,718,362
ST Rep B	210,907,864
ST Rep C	214,622,778
