# Supplementary material for: KLF4 recruits SWI/SNF to increase chromatin accessibility and reprogram the endothelial enhancer landscape under laminar shear stress
Source: Nat Commun. 2022 Aug 23;13:4941. doi: 10.1038/s41467-022-32566-9 (PMC9399231; doi:10.1038/s41467-022-32566-9)
Supplement: Supplementary file 2 — Reporting Summary [file 41467_2022_32566_MOESM2_ESM.pdf]

nature research

Corresponding author(s): Marlene Rabinovitch, MD

Last updated by author(s): Jul 28, 2022

Reporting Summary

Nature Research wishes to improve the reproducibility of the work that we publish. This form provides structure for consistency and transparency in reporting. For further information on Nature Research policies, see our Editorial Policies and the Editorial Policy Checklist.

Statistics

For all statistical analyses, confirm that the following items are present in the figure legend, table legend, main text, or Methods section.

n/a	Confirmed	
☐	☒	The exact sample size (*n*) for each experimental group/condition, given as a discrete number and unit of measurement
☒	☐	A statement on whether measurements were taken from distinct samples or whether the same sample was measured repeatedly
☐	☒	The statistical test(s) used AND whether they are one- or two-sided<br>*Only common tests should be described solely by name; describe more complex techniques in the Methods section.*
☒	☐	A description of all covariates tested
☐	☒	A description of any assumptions or corrections, such as tests of normality and adjustment for multiple comparisons
☐	☒	A full description of the statistical parameters including central tendency (e.g. means) or other basic estimates (e.g. regression coefficient) AND variation (e.g. standard deviation) or associated estimates of uncertainty (e.g. confidence intervals)
☐	☒	For null hypothesis testing, the test statistic (e.g. *F*, *t*, *r*) with confidence intervals, effect sizes, degrees of freedom and *P* value noted<br>*Give P values as exact values whenever suitable.*
☒	☐	For Bayesian analysis, information on the choice of priors and Markov chain Monte Carlo settings
☒	☐	For hierarchical and complex designs, identification of the appropriate level for tests and full reporting of outcomes
☐	☒	Estimates of effect sizes (e.g. Cohen's *d*, Pearson's *r*), indicating how they were calculated

Our web collection on statistics for biologists contains articles on many of the points above.

Software and code

Policy information about availability of computer code

Data collection	Base call files from Illumina sequencing were converted to fastq format using Bcl2fastq v2.17
Data analysis	ATAC-Seq was analyzed using the Kundaje Lab ATAC-Seq processing pipeline (https://github.com/kundajelab/atac_dnase_pipelines) with MACS2 v.2.1.2, DiffBind v2.4.8 and DESeq2 v1.20.0.<br>Motif enrichment analysis was performed with Homer v4.10.<br>For RNA-Seq analysis, reads were aligned to the GRCh37/hg19 genome using STAR v.2.5.4b, transcripts were quantitated using RSEM v.1.3.1 and differential analysis was performed with DESeq2 v1.20.0. Functional enrichment analysis was performed using Metascape.<br>ChIP-Seq was analyzed using the Kundaje Lab ChIP-seq processing pipeline (https://github.com/kundajelab/chipseq_pipeline) with DiffBind v2.4.8 and DESeq2 v1.20.0. ATAC-Seq and ChIP-Seq tracks were visualized using IGV Genome Browser v.2.8.0.<br>For AP-MS, .RAW data files were processed using Byonic v.2.14.27 (Protein Metrics) to identify peptides and infer proteins against the human Uniprot database.<br>For HiCHIP, data were filtered for duplicate reads, aligned to the GRCh37/hg19 genome and filtered for valid interactions using the HiC-Pro pipeline v.2.11.1 (https://github.com/nservant/HiC-Pro) using default settings. FitHiChIP (https://github.com/ay-lab/FitHiChIP) was used to determine significant interactions using default settings, with the exception of allowing interactions with a minimum size of 1Kb. The diffLoop package (https://github.com/aryeelab/diffloop) was used to test for differential interactions between conditions, and to infer enhancer-promoter relationships.<br>For ABC, ATAC-Seq and H3K27ac ChIP-Seq peaks were called using MACS2 v.2.1.2 The ABC v0.2 pipeline (https://github.com/broadinstitute/ABC-Enhancer-Gene-Prediction/) was used to identify enhancer promotor interactions. Candidate enhancer regions were defined by the ABC script makeCandidateRegions.py, which resized each peak to be 250 base pairs centered on the peak summit and counts ATAC-seq reads in each peak retaining the top 150,000 peaks with the most read counts. Blacklisted regions with known propensity for errors (hg19-blacklist.v2.bed from https://github.com/Boyle-Lab/Blacklist) were removed and any overlapping regions were merged. Enhancer activity was quantified by the ABC script run.neighborhoods.py, which counted ATAC-seq and H3K27ac ChIP-Seq reads in the candidate enhancer regions that were generated in the previous step, gene bodies, and promoter regions. Lastly, the ABC score was calculated using the ABC script

predict.py; which combined information from the enhancer and promoter activities, calculated in the previous step, with contact frequency data from average Hi-C profiles of 10 cell lines (ftp://ftp.broadinstitute.org/outgoing/lincRNA/average_hic/average_hic.v2.191020.tar.gz. The default threshold of 0.02 was applied which corresponds to approximately 70% recall and 60% precision (Fulco et al. Nat Genet. 2019).

For manuscripts utilizing custom algorithms or software that are central to the research but not yet described in published literature, software must be made available to editors and reviewers. We strongly encourage code deposition in a community repository (e.g. GitHub). See the Nature Research guidelines for submitting code & software for further information.

Data

Policy information about availability of data

All manuscripts must include a data availability statement. This statement should provide the following information, where applicable:

- Accession codes, unique identifiers, or web links for publicly available datasets
- A list of figures that have associated raw data
- A description of any restrictions on data availability

All raw sequencing data supporting the findings of this study have been deposited in the Gene Expression Omnibus (GEO). AP-MS data is deposited in ProteomeXchange. All other data supporting the findings of this study are available from the corresponding author on reasonable request.

Field-specific reporting

Please select the one below that is the best fit for your research. If you are not sure, read the appropriate sections before making your selection.

[x] Life sciences [ ] Behavioural & social sciences [ ] Ecological, evolutionary & environmental sciences

For a reference copy of the document with all sections, see nature.com/documents/nr-reporting-summary-flat.pdf

Life sciences study design

All studies must disclose on these points even when the disclosure is negative.

Sample size	No sample size calculations were performed. The sample size was determined based on our experience as well as reporting in literature. For ATAC-Seq n=3 independent experiments were performed, for RNA-Seq n=2 independent experiments, for AP-MS n=2 independent experiments. ATAC-qPCR and RT-qPCR were performed with at least n=3 experimental replicates as indicated in the figure legends. HiChIP was performed with n=3 experimental replicates, ABC analysis was from n=2 experimental replicates.
Data exclusions	No data were excluded from the study.
Replication	Experiments were performed multiple times to ensure that results are reproducible. All attempts at replication were succesful.
Randomization	All experiments were performed with cells obtained from donor control lungs and the tissue used for imaging was from healthy control rats, so no randomization was performed.
Blinding	Quantification of the immunofluorescent imaging including the PLA assays, was performed in a blinded manner, i.e. the investigators that analyzed the images were not aware of the experimental conditions from which these images were taken.

Reporting for specific materials, systems and methods

We require information from authors about some types of materials, experimental systems and methods used in many studies. Here, indicate whether each material, system or method listed is relevant to your study. If you are not sure if a list item applies to your research, read the appropriate section before selecting a response.

Materials & experimental systems

n/a	Involved in the study	
[ ]	[x]	Antibodies
[ ]	[x]	Eukaryotic cell lines
[x]	[ ]	Palaeontology and archaeology
[ ]	[x]	Animals and other organisms
[ ]	[x]	Human research participants
[x]	[ ]	Clinical data
[x]	[ ]	Dual use research of concern

Methods

n/a	Involved in the study	
[ ]	[x]	ChIP-seq
[x]	[ ]	Flow cytometry
[x]	[ ]	MRI-based neuroimaging

Antibodies

Antibodies used	For ChIP-Seq the following primary antibodies were used: H3K27ac (#8173, Cell signaling Technology, 1:100), H3K4me1 (#5326, Cell Signaling Technology, 1:50), KLF4 (sc20691, Santa Cruz Biotechnology, 1:120) and BRG1 (A303-877A, Bethyl Laboratories, 1:600).

For IF, primary antibodies were used targeting KLF4 (sc20691, Santa Cruz Biotechnology, 1:100), ATF2 (sc-242, Santa Cruz Biotechnology, 1:100) and ETS1 (sc55581, Santa Cruz Biotechnology, 1:100). Secondary antibodies were Alexa Fluor-conjugated donkey anti-mouse or donkey anti-rabbit antibodies (A-21206, 1:400 for KLF4; A-21203, 1:400 for ATF2; and A-31571, 1:400 for ETS1; all from Thermo Fisher Scientific).

For PLA in cultured cells, primary antibodies were used targeting KLF4 (sc20691, Santa Cruz Biotechnology, 1:100), and BRG1 (sc17796, Santa Cruz Biotechnology, 1:75), and SMARCC2 (sc17838, Santa Cruz Biotechnology, 1:75). For PLA in rat lung tissue, primary antibodies were used against KLF4 (sc20691, Santa Cruz Biotechnology, 1:50), and BRG1 (sc17796, Santa Cruz Biotechnology, 1:30), followed by post-staining with FITC-conjugated antibodies targeting aSMA (F377, Sigma-Aldrich, 1:400) and antibodies targeting vWF (ab6994, Abcam, 1:500) followed by Alexa Fluor 647-conjugated secondary antibodies (A32795, Thermo Fisher Scientific, 1:200).

Validation

All antibodies used in our study were validated by the respective commercial source for the application used. The H3K4me, H3K27ac and BRG1 antibodies used for ChIP-Seq have also been validated by the ENCODE project.

H3K27ac (D5E4): https://www.cellsignal.com/products/primary-antibodies/acetyl-histone-h3-lys27-d5e4-xp-rabbit-mab/8173; https://www.citeab.com/antibodies/125111-8173-acetyl-histone-h3-lys27-d5e4-xp-rabbit-mab.

H3K4me1 (D1A9): https://www.cellsignal.com/products/primary-antibodies/mono-methyl-histone-h3-lys4-d1a9-xp-rabbit-mab/5326; https://www.citeab.com/antibodies/125242-5326-mono-methyl-histone-h3-lys4-d1a9-xp-rabbit.

BRG1: https://www.bethyl.com/product/A303-877A/BRG1+SMARCA4+Antibody; https://www.citeab.com/antibodies/658317-a303-877a-brg1-smarca4-antibody.

KLF4 (H-180): https://www.citeab.com/antibodies/795644-sc-20691-gklf-antibody-h-180; https://www.citeab.com/antibodies/795644-sc-20691-gklf-antibody-h-180.

ATF2 (F2BR-1): https://www.scbt.com/p/atf-2-antibody-f2br-1; https://www.citeab.com/antibodies/805947-sc-242-atf-2-antibody-f2br-1.

ETS1 (C-4): https://www.scbt.com/p/ets-1-antibody-c-4; https://www.citeab.com/antibodies/792192-sc-55581-ets-1-antibody-c-4.

BRG1 (G-7): https://www.scbt.com/p/brg-1-antibody-g-7; https://www.citeab.com/antibodies/811862-sc-17796-brg-1-antibody-g-7.

SMARCC2 (E-6): https://www.scbt.com/p/baf170-antibody-e-6; https://www.citeab.com/antibodies/781662-sc-17838-baf170-antibody-e-6.

aSMA (1A4): https://www.sigmaaldrich.com/catalog/product/sigma/f3777; https://www.citeab.com/antibodies/2304934-f3777-monoclonal-anti-actin-smooth-muscle-fitc.

vWF: https://www.abcam.com/von-willebrand-factor-antibody-ab6994.html; https://www.citeab.com/antibodies/759548-ab6994-anti-von-willebrand-factor-antibody.

Eukaryotic cell lines

Policy information about cell lines

Cell line source(s)	Primary human pulmonary artery endothelial cells (PAEC) were either commercially obtained (PromoCell) or harvested from unused donor control lungs obtained though the Pulmonary Hypertension Breakthrough Initiative (PHBI). funded by NIH (R24 HL123767) and the Cardiovascular Medical Research and Education Fund (CMREF; UL 1RR024986).
Authentication	Cell lines were routinely checked for endothelial marker expression.
Mycoplasma contamination	Cells were routinely tested for mycoplasma contamination.
Commonly misidentified lines (See ICLAC register)	*Name any commonly misidentified cell lines used in the study and provide a rationale for their use.*

Animals and other organisms

Policy information about studies involving animals; ARRIVE guidelines recommended for reporting animal research

Laboratory animals	Healthy untreated surplus 8 week old Sprague Dawley rats (n=2)were sacrificed and lung tissues were processed for microscopic imaging studies.
Wild animals	The study did not involve wild animals.
Field-collected samples	The study did not involve samples from the field.

Ethics oversight	Procedures were compliant with all ethical regulations regarding animal research. Care and housing of the rats was in accordance with the guidelines from the Stanford University Administrative Panel on Laboratory Animal Care, and approved under APLAC protocol 31608.

Note that full information on the approval of the study protocol must also be provided in the manuscript.

Human research participants

Policy information about studies involving human research participants

Population characteristics	Human cells provided by the PHBI Initiative were obtained under the PHBI network IRB protocol, with informed consent and IRB approvals at the transplant procurement sites. The cell lines used were coded with no identifying information. Cells purchased from PromoCell were derived from tissues of donors who have signed an informed consent form. In both cases, the informed consent outlines in detail the purpose of the donation and the procedure for processing the tissue.
Recruitment	*Describe how participants were recruited. Outline any potential self-selection bias or other biases that may be present and how these are likely to impact results.*
Ethics oversight	*Identify the organization(s) that approved the study protocol.*

Note that full information on the approval of the study protocol must also be provided in the manuscript.

ChIP-seq

Data deposition

- [x] Confirm that both raw and final processed data have been deposited in a public database such as GEO.
- [x] Confirm that you have deposited or provided access to graph files (e.g. BED files) for the called peaks.

Data access links<br>*May remain private before publication.*	https://www.ncbi.nlm.nih.gov/geo/query/acc.cgi?acc=GSE152892 (token: wrkhuscefvgtjgx)<br>https://www.ncbi.nlm.nih.gov/geo/query/acc.cgi?acc=GSE152894 (token: urirgigupfsvpaj)
Files in database submission	H3K27ac.caMEK5.bw<br>H3K27ac.Control.bw<br>H3K4me1.caMEK5.bw<br>H3K4me1.Control.bw<br>KLF4.caMEK5.bw<br>KLF4.Control.bw<br>BRG1.caMEK5.bw<br>BRG1.Control.bw<br>caMEK5.INPUT.repA.R1<br>caMEK5.INPUT.repA.R2<br>caMEK5.INPUT.repB.R1<br>caMEK5.INPUT.repB.R2<br>GFP.INPUT.repA.R1<br>GFP.INPUT.repA.R2<br>GFP.INPUT.repB.R1<br>GFP.INPUT.repB.R2<br>caMEK5.H3K27ac.repA.R1<br>caMEK5.H3K27ac.repA.R2<br>caMEK5.H3K27ac.repB.R1<br>caMEK5.H3K27ac.repB.R2<br>GFP.H3K27ac.repA.R1<br>GFP.H3K27ac.repA.R2<br>GFP.H3K27ac.repB.R1<br>GFP.H3K27ac.repB.R2<br>caMEK5.H3K4me1.repA.R1<br>caMEK5.H3K4me1.repA.R2<br>caMEK5.H3K4me1.repB.R1<br>caMEK5.H3K4me1.repB.R2<br>GFP.H3K4me1.repA.R1<br>GFP.H3K4me1.repA.R2<br>GFP.H3K4me1.repB.R1<br>GFP.H3K4me1.repB.R2<br>caMEK5.KLF4.repA.R1<br>caMEK5.KLF4.repA.R2<br>caMEK5.KLF4.repB.R1<br>caMEK5.KLF4.repB.R2<br>GFP.KLF4.repA.R1

GFP.KLF4.repA.R2
GFP.KLF4.repB.R1
GFP.KLF4.repB.R2
caMEK5.DS_INPUT.repA.R1
caMEK5.DS_INPUT.repA.R2
caMEK5.DS_INPUT.repB.R1
caMEK5.DS_INPUT.repB.R2
GFP.DS_INPUT.repA.R1
GFP.DS_INPUT.repA.R2
GFP.DS_INPUT.repB.R1
GFP.DS_INPUT.repB.R2
caMEK5.BRG1.repA.R1
caMEK5.BRG1.repA.R2
caMEK5.BRG1.repB.R1
caMEK5.BRG1.repB.R2
GFP.BRG1.repA.R1
GFP.BRG1.repA.R2
GFP.BRG1.repB.R1
GFP.BRG1.repB.R2

Genome browser session
(e.g. UCSC)

https://genome.ucsc.edu/s/jrmoonen/NCB_Moonen_et_al._Fig.3d
https://genome.ucsc.edu/s/jrmoonen/NCB_Moonen_et_al._Fig.5b
https://genome.ucsc.edu/s/jrmoonen/NCB_Moonen_et_al._Fig.5c
https://genome.ucsc.edu/s/jrmoonen/NCB_Moonen_et_al._Fig.6b
https://genome.ucsc.edu/s/jrmoonen/NCB_Moonen_et_al._Fig.6c

Methodology

Replicates

Two experimental replicates were used for all ChIP-Seq studies.

Sequencing depth

Samples (merged) Peak count

H3K27ac.caMEK5 68,069
H3K27ac.GFP 62,759

H3K4me1.caMEK5 121,021
H3K4me1.GFP 132,032

KLF4.caMEK5 66,100
KLF4.GFP 6,076

BRG1.caMEK5 60,652
BRG1.GFP 49,653

Antibodies

H3K27ac (D5E4; #8173, Cell Signaling)
H3K4me1 (D1A9; #5326, Cell Signaling)
BRG1 (A303-877A, Bethyl Laboratories)
KLF4 (H-180; sc-20691, Santa Cruz Biotechnology)

Peak calling parameters

Data were processed using the Kundaje Lab ChIP-seq processing pipeline (https://github.com/kundajelab/chipseq_pipeline). Peaks were called using MACS2 with p 1e-5

Data quality

Samples (merged repl.)	Peak count (FDR10%)
H3K27ac.caMEK5	68,069
H3K27ac.GFP	62,759
H3K4me1.caMEK5	121,021
H3K4me1.GFP	132,032
KLF4.caMEK5	66,100
KLF4.GFP	6,076
BRG1.caMEK5	60,652
BRG1.GFP	49,653

Software

R v.3.5.0, MACS2 v.2.1.2, DiffBind v2.4.8 and DESeq2 v1.20.0.
